# Polydextrose Enhances Calcium Absorption and Bone Retention in Ovariectomized Rats

**DOI:** 10.1155/2013/450794

**Published:** 2013-05-25

**Authors:** Adriana R. Weisstaub, Victoria Abdala, Macarena Gonzales Chaves, Patricia Mandalunis, Ángela Zuleta, Susana Zeni

**Affiliations:** ^1^Food Science and Nutritional Department, School of Pharmacy and Biochemistry, Buenos Aires University (UBA), 1114 Buenos Aires, Argentina; ^2^Metabolic Bone Diseases Laboratory, Clinical Hospital, Immunology, Genetic and Metabolism Institute (INIGEM), National Council for Scientific and Technologic Research (CONICET), UBA, 1114 Buenos Aires, Argentina; ^3^Histologycal and Embryology Department, School of Dentistry, UBA, 1114 Buenos Aires, Argentina

## Abstract

*Purpose. *To evaluate the effect of polydextrose (PDX) on Ca bioavailability and prevention of loss of bone mass. *Methods.* Twenty-four two-month-old ovariectomized rats were fed three isocaloric diets only varied in fiber source and content up to 60 days (FOS group, a commercial mixture of short- and long-chain fructooligosaccharide, OVX group fed AIN 93 diet, and PDX group). A SHAM group was included as control. Apparent Ca absorption percentage (%ABS), changes in total skeleton bone mineral content (tsBMC) and bone mineral density (BMD) and femur BMD, % Bone Volume, Ca and organic femur content, caecal weight, and pH were evaluated. *Results.* %ABS and caecum weight of PDX and FOS were higher, and caecum pH was lower compared to OVX and SHAM. PDX reached a higher pH and lower caecum weight than FOS possibly because PDX is not completely fermented in the colon. Changes in tsBMC and femur BMD in FOS and PDX were significant lower than SHAM but significantly higher than OVX. % Bone Volume and femur % of Ca in PDX were significantly higher than OVX and FOS but lower than SHAM. *Conclusions*. PDX increased Ca absorption and prevented bone loss in OVX rats.

## 1. Introduction

 Although an optimal calcium intake (CaI) is vital throughout the life cycle, a great percentage of population consumes levels that are far below the recommended amounts. Ca nutrition adequacy is necessary for both bone accretion during growth to achieve an optimum peak bone mass and maintenance in the adult life to suppress bone turnover and, therefore, bone loss [1, 2]. Bone health requires an adequate consumption of Ca to maintain its homeostasis. When Ca levels decrease, parathyroid hormone (PTH) is released to increase bone resorption and to indirectly absorb the available Ca to elevate levels of serum Ca to normal ranges. 

Although the increment in CaI would be the most effective strategy for avoiding Ca deficit, if the intake remains inadequate, the ability to improve absorption becomes an important tool to optimize bone health. Ca absorption average only 30% in the adult and, regardless of the CaI, losses through endogenous secretions approximate 120 mg/d [3]. Most of the Ca absorption occurs in the small intestine; however, if the mineral remains ionized and in solution, about 5% occurs in the colon. Several dietary factors could enhance Ca absorption. In this regard, the solubilisation of Ca salts by the acids generated through microbial fermentation in the large intestine has been proposed as one of the mechanisms responsible for the increase in Ca absorption observed following ingestion of highly fermentable, indigestible materials [4]. 

 Nondigestible oligosaccharides can modify the colonic microbiota by increasing the proliferation and activity of beneficial flora [5] which induces changes in the enzymatic activity and produces compounds that enhance paracellular and transcellular absorption of Ca [6]. Several type fructans compounds including inulin, oligofructose, or a mixture of short- and long-chain products (Synergy) [7] have been investigated related to their effect on Ca absorption and bone health [8, 9]. 

 Fiber is an essential nutrient in a healthy diet, contributing to health maintenance and preventing the occurrence of different diseases. Polydextrose (PDX), a compound widely used as an additive for more than 15 years, is a soluble fiber, that is, not digested in the upper gastrointestinal tract [10, 11]. The beneficial effect on gastrointestinal function of PDX has been largely studied; however, few reports were carried out regarding the effect on Ca absorption and bone retention, and several of them were done in normal or gastrectomized rats [4].

 The adult ovariectomized (OVX) rat is a suitable animal model to mimic the estrogen withdrawal of postmenopausal women [12, 13]. Bone loss by estrogen withdrawal is a well-known consequence of OVX. As the increment of CaI decreases bone turnover, a recommended manner to prevent bone loss would be increasing the Ca absorption. 

 On these bases, the aim of the present report was to evaluate the effect of feeding an adequate Ca diet containing 5 g/kg of PDX on Ca absorption and retention into bone in young-OVX rats. In addition, the effect of this diet in preventing the loss of bone mass in OVX rats was compared with the same Ca diet containing a commercial mixture of short- and long-chain fructo-oligosaccharide (FOS) products (Synergy) whose effects on these studied parameters are well documented.

## 2. Methods and Materials 

### 2.1. Rats and Diets

 A total of 36 two-month-old female virgin Wistar rats (195.0 ± 9.0 g) were obtained from the Laboratory Animal Service of the School of Biochemistry and Pharmacy, Buenos Aires University (Argentina). Throughout the experiment, animals were allowed free access to deionized water and food and were housed in individual stainless steel cages in a temperature- (21 ± 1°C) and humidity- (60 ± 10%) controlled room with a 12 h light/dark cycle. They were fed a commercial stock diet for laboratory rats during 7 days for acclimatization (Gavave SA, Argentina). After that, ovariectomy was performed by a dorsal approach under light anesthesia (0.1 mg/100 g body weight (BW) of ketamine hydrochloride + 0.1 mg/100 g BW of acepromazine maleate) (Holliday-Scott S A, Buenos Aires, Argentina) and randomly placed on one of the 3 following diets (*n* = 8/group) during a 60-day period (Table 1):

- SHAM groups: rats were fed a semisynthetic diet prepared according to the American Institute of Nutrition Diet (AIN 93 M) [14];
- OVX group: rats were fed a semisynthetic diet prepared according to the American Institute of Nutrition Diet (AIN 93 M);
- FOS group (FOS): rats were fed the AIN 93 M diet containing 10 g/100 g diet of Synergy (Orafti BENEO, Tienen, Belgium) replacing equal amounts of cornstarch and sucrose;
- PDX group (PDX): rats were fed the AIN 93 M containing 5 g/100 g diet of polydextrose (Litesse^R^, Danisco) replacing equal amounts of cornstarch and sucrose.

Diets were isocaloric and supplied a similar amount of Ca (0.5%) and phosphorus (P) (0.3%).

 BW was recorded once a week throughout the study. Food intakes were recorded every three days throughout the experiment, and the total intake, daily intake, and daily CaI were then calculated. This study was carried out in accordance with the National Institute of Health Guide for the Care and Use of Laboratory Animals and approved by the Committee of Health Guide for the Care and Use of Laboratory Animals of the Facultad de Farmacia y Bioquímica, Universidad de Buenos Aires. 

### 2.2. Apparent Calcium Absorption

Food intake and feces recorded during the last three days of the experiment were used to calculate the apparent Ca absorption (%ABS) as follows: [(CaI − Fecal Ca)/CaI] × 100. 

### 2.3. Bone Measurements

At the beginning (*t* = 0) and at the end of the experiment (*t* = 60), total skeleton bone mineral content (tsBMC) and bone mineral density (tsBMD) were determined “in vivo” under light anesthesia with a total body scanner by dual energy X-ray absorptiometry (DXA) provided with a specifically designed software for small animals (DPX Alpha, Small Animal Software, Lunar Radiation Corp., Madison, WI, USA) as previously described [15]. In brief, all rats were scanned using an identical scan procedure. Precision was assessed by measuring one rat five times with repositioning between scans on the same and on different days. The coefficient of variation (CV) was 0.9% for total skeleton BMD and 3.0% for BMC. The analysis of the different subareas was carried out on the image of the animal on the screen using a ROI for each segment. The BMD CV was 2.2% for the femur. To minimize the interobserver variations, all analyses were carried out by the same technician. To avoid possible differences in BMC for a different BW, the data of BMC were expressed as percentage of BW.

At the end of the experience, rats were placed under anesthesia (0.1 mg/100 g body weight of ketamine hydrochloride + 0.1 mg/100 g BW of acepromazine maleate), and the right femur was excised at sacrifice for biochemical analysis. The right tibia was resected and fixed by immersion in buffered formalin for 48 h, decalcified in 10% ethylene-diamine tetraacetic acid (EDTA) (pH 7) during 25 days, and embedded in paraffin. An 8- to 10-*μ*m-thick longitudinally oriented section of subchondral bone was obtained at the level of the middle third, including primary and secondary spongiosa. It was stained with haematoxylin-eosin and microphotographed (AXIOSKOP, Carl Zeiss) to perform bone volume % (BV%) on the central area of the metaphyseal bone displayed on the digitalized image [16].

### 2.4. Caecal pH

 After the rats were killed, the caecum from each rat was excised, weighed, and split open, and the pH recorded. 

### 2.5. Analytical Procedures

Feces were dried under infrared light and pounded. Diets and feces were wet ashes with nitric acid using Parr bombs [17]. 

Femurs were cleaned of any adhering soft tissue and dried at 100°C for 72 hours, and fat was extracted by immersion for 15 days in chloroform-methanol (3 : 1) mixture which was removed and replaced every three days. Finally, it was dried for 48 hours at 100°C. The fat-free and dried femurs were weighed and ashes were obtained at 700°C until white and crystalline. Thereafter, they were dissolved in HCl and diluted for Ca and P analysis. The amounts of Ca and P were calculated as total content and percentage content of dried fat-free tissue, and the femur Ca/P ratio was also calculated. 

 Ca concentration in diets, feces, and femur was determined using an atomic absorption spectrophotometer [18]. Lanthanum chloride (6500 mg/L in the final solution) was added to avoid interferences. P concentrations were measured according to the Gomori method [19]. NIST reference material RM 8435 (whole milk powder) was also subjected to identical treatment to verify accuracy of the analytical procedures and treated with each bath of samples to ensure accuracy and reproducibility of mineral analysis. 

### 2.6. Statistical Analysis

Results were expressed as mean ± standard deviation (SD). Differences were tested by one-way analysis of variance (ANOVA) using the INSTAT package. When ANOVA presented statistical differences (*P* < 0.05), intragroup comparisons were tested by Tukey test.

## 3. Results

### 3.1. Food Consumption, Calcium Intake, and Body Weight Gain

 As expected, Table 2 shows a lower food consumption (g/60 days), daily diet intake (g/day), daily CaI (mg/day), and BW gain (g/60 days) in SHAM group as compared to the 3 OVX groups which did not present differences among them.

**Figure 1:**
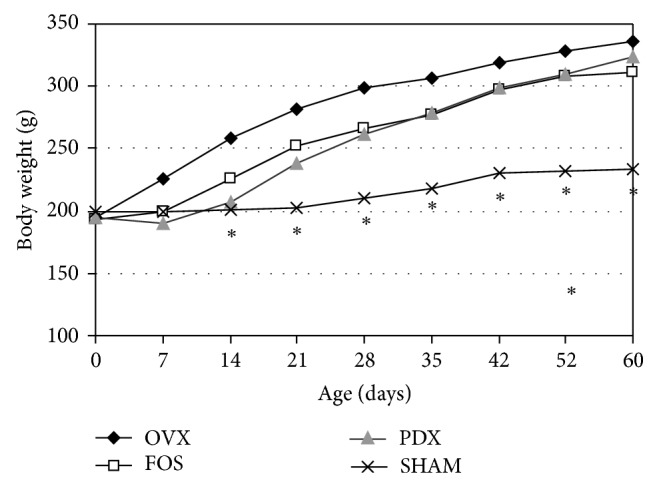
Body weight (g) throughout the study. ∗*P* < 0.01 versus the other three studied groups. Differences were analyzed by Bonferroni test after ANOVA.

Figure 1 showed the BW throughout the experiment. BW was significantly lower in SHAM group as compared to OVX, FOS, and PDX groups (*P* < 0.01) throughout the study; BW was also significantly lower in OVX versus FOS and PDX groups from the 1st to the 5th week of the study (*P* < 0.01) without differences between them. Thereafter, no significant differences among the 3 OVX groups were found until the end of the experiment (8th week). During the studied period, the BW gain was higher in the 3 OVX groups versus SHAM group (*P* < 0.05); no differences between PDX and FOS groups were observed which showed a tendency to reach lower values as compared to OVX groups (Table 2). 

### 3.2. Caecum pH and Weight and Calcium Absorption

The Ca fecal excretion was significantly lower, and the %ABS was significantly higher in PDX and FOS as compared to OVX and SHAM groups (*P* < 0.01) without differences between them; in addition, no differences were observed between OVX and SHAM groups while caecum pH was higher and caecum weight was lower in PDX versus FOS group (*P* < 0.01) (Table 3). 

### 3.3. Bone Mineral Content and Density, Femur Composition, and Bone Volume

Total skeleton BMC (tsBMC) and BMD (tsBMD) and their changes between the end and the beginning of the study are shown in Table 4. No significant differences were observed at *t* = 0 among groups; however, tsBMC at *t* = 60 was lower in the 3 OVX groups as compared to SHAM group (*P* < 0.05) without differences between them. The changes in BMC between *t* = 60 and *t* = 0 of FOS and PDX groups were significantly lower than SHAM group (*P* < 0.05) but significantly higher than OVX group, expressed as total content (*P* < 0.01) or normalized per BW (*P* < 0.01) without significant differences between them. The tsBMD and its changes were higher in SHAM than in OVX groups (*P* < 0.05) and in FOS and PDX than in OVX group (*P* < 0.01) without differences between them. Femur BMD (fBMD) changes were significantly lower in OVX groups as compared to SHAM group (*P* < 0.05) and significantly higher in FOS and PDX than in OVX group (*P* < 0.05), without differences between them. 

Table 5 shows right femur ashes and organic content and femoral Ca and P content at the end of the experiment. All these parameters were significantly lower in OVX than in SHAM group (*P* < 0.05), and only ashes content reached significance as compared to FOS and PDX groups (*P* < 0.05) which did not present significant differences between them, but they were also lower than SHAM group (*P* < 0.05). The ashes/organic content ratio was higher in SHAM than in the 3 OVX groups (*P* < 0.05) which did not present differences among them while no significant differences were observed in femoral Ca/P ratio.

**Figure 2:**
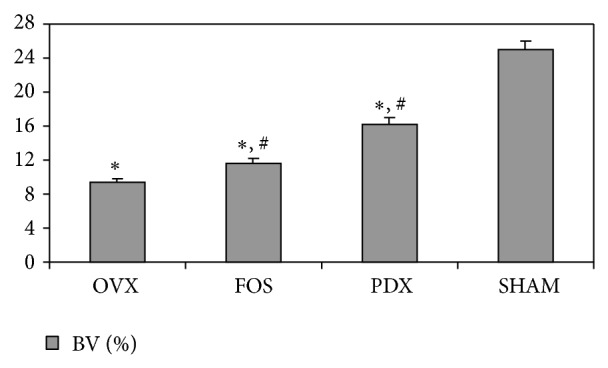
Femoral bone volume percentage (BV%) at the end of the study. ∗*P* < 0.01 versus SHAM; ^#^ *P* < 0.05 versus OVX. Differences were analyzed by Bonferroni test after ANOVA.

In agreement with BMD and femoral Ca content, the highest bone volume % was observed in SHAM group (*P* < 0.01); in addition, PDX and FOS presented a significantly higher value than OVX group (*P* < 0.05), and there was a tendency to be higher in PDX than in FOS group without reaching significance (Figure 2). 

## 4. Discussion

The results of the present report showed that OVX rats feeding a diet according to AIN 93 M that contains a 5% of PDX during 60 days increased Ca absorption and bone Ca content. 

When food intake and BW were compared, the data showed that no differences among OVX groups were observed confirming that PDX diet did not impair diet consumption or BW gain. In addition, as the diet had the same Ca content, no differences in CaI were observed; however, faecal Ca was lower in both DPX and FOS groups. As a result, PDX Ca absorption was as higher as that observed in the FOS-containing diet. Moreover, bone Ca bioavailability was also enhanced in the PDX diet as observed by the increment in BMC, femoral Ca content, and bone volume. 

Ca absorption and bioavailability depend not only on luminal Ca concentration, but also on age. Ca absorption in normal rats feeding the recommended dietary Ca levels reached the highest levels at weaning and decreased thereafter to reach the lowest values in adult life [20]. All the OVX studied rats herein were of similar age and fed diets containing the same level of Ca; then, Ca absorption may have been affected by the fiber source and content. The mechanism of Ca absorption involves active, saturable, transcellular movement that takes place largely in the duodenum and passive, nonsaturable, paracellular movement that takes place throughout the small intestine. The sojourn time that a soluble ion remains in the small intestine segments determines how much it is absorbed in each segment. Ca duodenum sojourn time is a matter of minutes, whereas in the lower part of the small intestine is about 2 hr in people and 3 hr in rats [21, 22]. The Ca absorption percentage is about 30%, depending on nutritional status, type, and content of dietary Ca (dairy products versus others), bioavailability, physiological status, and so forth. [21, 22]. Then, a significant more Ca could be potentially absorbed if the insoluble, unabsorbed Ca coming from the small intestine is maintained to an anionic form in the colon. 

Soluble fibers and oligosaccharides are being investigated for their potential to improve bone health largely through their influence on mineral absorption and retention. The beneficial effect of inulin, a long-chain fructo-oligosaccharide (FOS) often obtained from chicory root, and other FOSs has been extensively studied in several experimental models [23]. In this regard, the effect on Ca metabolism and bone health of different substances, such as inulin, oligofructose, or a mixture of short- and long-chain products (Synergy) was tested [7]. Although FOSs enhance Ca absorption, only inulin-FOS increases Ca retention in rats [24, 25]. PDX is a soluble fiber that could be fermented in the gut with production of short-chain fatty acids (SCFA) and reduction of pH. Several studies showed that PDX increases Ca absorption and retention [26, 27]. Mineo et al. [26] “in vitro” found that PDX enhances net Ca transport from the small and large intestine epithelium of rats. Hara et al. and dos Santos et al. [4, 27] demonstrated that the ingestion of PDX increases Ca absorption in normal and total gastrectomized rats suggesting that the small intestine rather than the large one is the responsible for Ca absorption increase. However, no studies were done to demonstrate PDX effect on OVX rats in which an extra Ca supplied may be relevant for avoiding bone mass diminution.

In the present paper the source of fiber in the 3 diets was different. While cellulose was the fiber source of the diet fed by the OVX and SHAM groups (AIN 93 M diet), FOS and PDX diets contained a 10% and 5% of different soluble fibers, a mixture of short- and long-chain products (Synergy) and PDX, respectively. It is important to point out that we performed a preliminary test with a diet containing 10% PDX; however, such diet was not well tolerated by the rats. One possible explanation for this effect would be that PDX is not fully fermented in the colon which induced intolerance and diarrhea. Synergy and PDX induce changes in the intestinal microflora which produces SCFA by fermentation that decrease caecal pH [28]. The low pH maintains Ca and other minerals in solution which improves their absorptions [23, 29–31]. Furthermore, it is also known that the rate and degree of fermentation and, consequently, the acidity could vary with the type and concentration of polysaccharide. According to the literature, the colonic fermentation range for various fibers is broad, from approximately 5% for cellulose to nearly complete for pectin [32]. In the present report, a reduction in the caecal pH was observed in the diets containing PDX or FOS; however, PDX groups showed a low pH reduction as compared to FOS. This effect may be partially explained by the fact that PDX is not completly fermented in the colon; as a consequence, it may also induce a lower intestinal wall thickening leading to the lower caecum weight than was also observed in PDX. 

 SCFA directly or indirectly participated in Ca absorption. In this regard, Ca acetate and propionate pass across cell membranes even more readily than ionized Ca alone [33]. In addition, butyrate improves the active and passive absorption of Ca. Indeed, butyrate is a potent stimulator of CaBP-9 kDa expression involved in the active Ca absorption [34]. In addition, the passive transepithelial absorption via the activation of tight junctions can be also promoted by FOS and PDX [26, 35]. Therefore, the greatest impact on mineral utilization would depend on the greatest SCFA production and consequently pH lowering. Nevertheless, the mechanism of enhanced Ca absorption with PDX supplementation has been debated. Some authors found a decreased of pH and an increment of SCFA production but others not. Oliveira et al. [36] observed that the addition of PDX to fermented milk produced the highest postacidification when compared to maltodextrin and oligofructose. Mäkeläinen et al. [37], working with a 4-stage colon simulator, founded an increment of the concentrations of all SCFA, especially acetate and propionate. In humans, Hengst et al. [38] showed a decrease on pH value of fecal content but did not find changes on SCFA concentrations. Conversely, other authors [39–41] “in vitro” found that PDX alone or mixed with other FOS lowered gas production rates, butyrate amount, and total SCFA production. In the present report, SCFA production was not measured; however, data showed a significant reduction in caecal pH and a caecum content twice higher than that observed in OVX group confirming the good fermentability of PDX diet [42]. In addition, despite the differences observed in these parameters when PDX and FOS groups were compared, no differences in Ca absorption were found between them. These results are in agreement with a previous report showing that Ca absorption was increased in rats feeding an inulin-FOS diet despite an increment in the total fecal mass [29]. Several papers in OVX rats had observed an increment in Ca absorption by feeding a diet containing 5% FOS and the double of Ca (1%) used in present study [43] or with the same Ca content (0.5%) but a higher (10%) FOS level [7, 32, 43]. In the present study, a concentration of 50 g PDX/kg diet was used because, as previously mentioned, a higher concentration was no well tolerated by the animals. Nevertheless, Ca absorption was higher than OVX groups and not different from FOS diet indicating a stimulating effect on Ca absorption by PDX.

The amount of Ca absorption and bone retention depends on several experimental conditions such as age, sex, hormonal status, duration of experiment, and diet composition including level of Ca and fructooligosaccharides. Although the absorption measurements could only evidence changes on Ca metabolism for a short time, determinations such as Ca in bone, bone density, and/or histology results showed a long-term impact. In this regard, it has been shown that inulin-type fructans, under certain conditions including OVX, stimulate Ca absorption and enhance bone Ca content [8, 9, 28, 44], although results were conflicting. In this regard, Scholz-Ahrens and Schrezenmeir [8] found that 5% of oligofructose given with the recommended amount of Ca was too low to stimulate Ca absorption and retention in adult OVX rats. The authors explained their findings suggesting a decrease in intestinal and renal functions associated with the rats aging. Conversely, Zafar et al. [25] found that 5.5% of Synergy in a normal Ca diet had positive effects on Ca absorption and retention in OVX rats. Moreover, Taguchi et al. [44] demonstrated an increment in trabecular bone, although Ca absorption was unaffected by feeding a diet containing 5% FOS and 0.5% Ca. The results of the present report showed that Ca absorption was improved in both AIN 93 M diets containing 5% PDX or 10% FOS supplying an extraamount of Ca to maintain homeostasis reducing Ca bone resorption and bone loss. Indeed, under our experimental conditions, bone Ca loss induced by OVX was partially prevented by feeding the PDX and FOS diets evidenced by the higher bone volume, femur ashes content, and tsBMC. Moreover, although no changes in tsBMD were observed, the BMD showed an increase at the femur site. Such differences can be explained taking into account that the total skeleton has a great percentage of cortical bone, while femur and proximal tibia sites have a higher percentage of trabecular bone, metabolic more active and consequently more susceptible to changes in bone remodeling.

Although further studies are required, the results of the present report demonstrated that PDX has a prebiotic effect. Moreover, this ingredient given jointly with an adequate amount of Ca optimizes its absorption and bone retention in OVX rats improving bone health. This effect may have important implications in preventing osteoporosis.

## Figures and Tables

**Table 1:** Composition of the experimental diets.

Ingredient (g/100 g diet)	AIN 93 M diet	FOS diet	PDX diet
Protein∗	12.0	12.0	12.0
Lipids∗∗	4.0	4.0	4.0
Mineral mix∗∗∗	3.5	3.5	3.5
Vitamin mix∗∗∗∗	1.0	1.0	1.0
L-Cystine	0.18	0.18	0.18
Choline	0.15	0.15	0.15
Cellulose	5.0		
Synergy^#^		10.0	
Polydextrose^##^			5.0
Dextrin	To complete 100 g	To complete 100 g	To complete 100 g

^*^Potassum Caseinate, Nestlé Argentina S.A, containing 85.1% of protein and 0.095 g% Ca.

^**^Commercial soy oil.

^***^Composition according AIN 93 M-MX.

^****^Composition according AIN 93-VX.

^#^Orafti BENEO, Tienen, Belgium.

^##^Litesse^R^, Danisco.

Dextrin was added as carbohydrate source to achieve 100 g of diet.

**Table 2:** Food consumption throughout the study, daily intake, daily Ca intake (Ca I) and body weight (BW) gain.

	Intake (g/60 days)	Daily intake (g/day)	Ca I (mg/day)	BW gain (g/60 days)
SHAM	**967.6 **±** 45.3**	**14.8 **±** 0.1**	**77.0 **±** 1.1**	**95.2 **±** 22.4**
OVX	1073.8 ± 23.4^*^	16.6 ± 0.3^*^	83.1 ± 1.3^*^	143.0 ± 31.2^*^
FOS	987.0 ± 96.3^*^	15.7 ± 1.4^*^	78.3 ± 6.9^*^	129.4 ± 27.3^*^
PDX	1022.9 ± 109.4^*^	16.4 ± 1.7^*^	81.9 ± 8.8^*^	137.1 ± 33.4^*^

Data are expressed as mean ± SD.^*^ *P* < 0.05 versus SHAM. Differences among groups were analyzed by Bonferroni test after ANOVA.

**Table 3:** Daily fecal Ca excretion, percentage of apparent Ca absorption (% ABS) during the last three days of the experiment, and caecum weight and caecum pH at the end of the study.

	Fecal Ca (mg/day)	% ABS	Caecum weight (g)	Caecum pH
SHAM	**39.9 ± 17.2**	**52.3 ± 6.1**	**2.51 ± 0.37**	**7.06 ± 0.16**
OVX	45.6 ± 14.0^*^	43.2 ± 5.5^*^	2.40 ± 0.45	7.06 ± 0.19
FOS	28.0 ± 8.7^∗,∗∗^	61.6 ± 4.9^∗,∗∗^	6.05 ± 0.59^∗,∗∗^	5.77 ± 0.21^∗,∗∗^
PDX	28.1 ± 10.3^∗,∗∗^	58.0 ± 4.1^∗,∗∗^	4.13 ± 0.84^∗,∗∗,#^	6.61 ± 0.18^∗,∗∗,#^

% Abs was determined during the last three days of the experience. Data are expressed as mean ± SD. ^*^ *P* < 0.01 versus SHAM; ^**^ *P* < 0.01 versus OVX and ^#^ *P* < 0.05 versus FOS. Differences among groups were analyzed by Bonferroni test after ANOVA.

**Table 4:** Total skeleton bone mineral content (tsBMC) (expressed as mg and as mg/g BW); total skeleton bone mineral density (tsBMD) and femur BMD (fBMD) at the beginning (*t* = 0) and at the end of the study (*t* = 60) and their respective changes between *t* = 60 and *t* = 0.

	SHAM	OVX	FOS	PDX
tsBMC *t* = 0 (mg)	**2936 ± 431**	3125 ± 630	2643 ± 312	2759 ± 265
tsBMC *t* = 60 (mg)	**6228 ± 304**	5410 ± 416^*^	5517 ± 659^*^	5947 ± 592^*^
tsBMC *t* = 60–tBMC *t* = 0 (mg)	**4587 ± 216**	2285 ± 214^*^	3096 ± 611^∗,∗∗^	3189 ± 472^∗,∗∗^
tBMC *t* = 0 (mg/g BW)	**13.8 ± 1.6**	14.2 ± 2.6	13.9 ± 1.6	14.2 ± 1.5
tBMC t = 60 (mg/g)	**19.7 ± 2.1**	16.0 ± 2.6^*^	18.2 ± 3.1^∗,#^	18.1 ± 2.6^∗,#^
tsBMC t = 60–tBMC *t* = 0 (mg/g)	**6.5 ± 1.4**	1.33 ± 0.04^*^	5.23 ± 3.55^∗,∗∗^	4.48 ± 2.41^∗,∗∗^
tsBMD *t* = 0 (mg/cm^2^)	**248.2 ± 2.1**	251.0 ± 5.5	247.7 ± 2.6	247.6 ± 8.0
tsBMD *t* = 60 (mg/cm^2^)	**281.3 ± 1.9**	278.5 ± 2.7	274.0 ± 7.5	278.1 ± 5.0
tBMD *t* = 60–tBMD *t* = 0 (mg/cm^2^)	**34.2 ± 2.2**	27.5 ± 2.7^*^	29.4 ± 1.9^∗,#^	30.7 ± 8.8^∗,#^
fBMD *t* = 0 (mg/cm^2^)	**235.3 ± 25.8**	232.0 ± 35.4	231.5 ± 17.0	227.7 ± 9.6

fBMD *t* = 60 (mg/cm^2^)	**298.3 ± 10.9**	253.5 ± 13.4^*^	278.7 ± 19.7^*^	272.0 ± 9.3^*^

fBMD *t* = 60–fBMD *t* = 0 (mg/cm^2^)	**62.1 ± 10.8**	21.5 ± 17.0^*^	47.2 ± 11.1^∗,∗∗^	44.3 ± 14.6^∗,∗∗^

Data are expressed as mean ± SD. ^*^ *P* < 0.05 versus SHAM group; ^#^ *P* < 0.05, ^**^ *P* < 0.01 versus OVX group. Differences were analyzed by Bonferroni test after ANOVA.

**Table 5:** Ashes and organic femur content; ashes/organic ratio, Ca and P femur content and femur Ca/P ratio.

	SHAM	OVX	FOS	PDX
Ashes content (mg/100 g BW)	**72.3 ± 2.4**	44.1 ± 2.5^*^	51.2 ± 11.5^∗,#^	54.8 ± 1.9^∗,#^
Organic content (mg/100 g BW)	**74.4 ± 1.2**	55.9 ± 2.4^*^	57.2 ± 1.5^*^	55.2 ± 1.9^*^
Ashes/Organic ratio (mg/mg)	**0.98 ± 0.22**	0.79 ± 0.08^*^	0.89 ± 0.04^#^	0.93 ± 0.16^#^
Femoral Ca content (mg/100 g BW)	**29.9 ± 3.6**	15.4 ± 4.1^*^	18.7 ± 2.2^∗,#^	20.4 ± 1.2^∗,#^
Femoral P content (mg/100 g BW)	**15.1 ± 3.3**	7.6 ± 1.6^*^	9.5 ± 1.1^∗,#^	10.2 ± 1.2^∗,#^
Femoral Ca/P ratio (mg/mg)	**2.01 ± 0.11**	2.00 ± 0.12	2.07 ± 0.20	2.11 ± 0.25

Femur parameters were determined at the end of the experience. Data are expressed as mean ± SD. ^*^ *P* < 0.05 versus SHAM group; ^*^ *P* < 0.05 versus SHAM. Differences were analyzed by Bonferroni test after ANOVA.
